# Cerebral perfusion pressure, microdialysis biochemistry and clinical outcome in patients with traumatic brain injury

**DOI:** 10.1186/1756-0500-4-540

**Published:** 2011-12-14

**Authors:** Theoniki Paraforou, Konstantinos Paterakis, Konstantinos Fountas, George Paraforos, Achilleas Chovas, Anastasia Tasiou, Maria Mpakopoulou, Dimitrios Papadopoulos, Antonios Karavellis, Apostolos Komnos

**Affiliations:** 1 General Hospital of Larissa, Larissa, Greece; 2 Assistant Professor, Department of Neurosurgery, University Hospital of Larissa, Larissa, Greece; 3 Department of Neurosurgery, University Hospital of Larissa, Larissa, Greece; 4 General Hospital of Larissa, Larissa, Greece; 5 Professor Εmeritus, Department of Neurosurgery, University Hospital of Larissa, Larissa, Greece; 6 Institute of Biomedical Research and Technology (BIOMED)/CERETETH, 51 Papanastasiou Str, 41222 Larissa, Greece

**Keywords:** Traumatic brain injury, Microdialysis, Cerebral perfusion pressure, Outcome, Intensive care unit

## Abstract

**Background:**

Traumatic Brain Injury (TBI) is a major cause of death and disability. It has been postulated that brain metabolic status, intracranial pressure (ICP) and cerebral perfusion pressure (CPP) are related to patients' outcome. The aim of this study was to investigate the relationship between CPP, ICP and microdialysis parameters and clinical outcome in TBIs.

**Results:**

Thirty four individuals with severe brain injury hospitalized in an intensive care unit participated in this study. Microdialysis data were collected, along with ICP and CPP values. Glasgow Outcome Scale (GOS) was used to evaluate patient outcome at 6 months after injury. Fifteen patients with a CPP greater than 75 mmHg, L/P ratio lower than 37 and Glycerol concentration lower than 72 mmol/l had an excellent outcome (GOS 4 or 5), as opposed to the remaining 19 patients. No patient with a favorable outcome had a CPP lower than 75 mmHg or Glycerol concentration and L/P ratio greater than 72 mmol/l and 37 respectively. Data regarding L/P ratio and Glycerol concentration were statistically significant at *p *= 0.05 when patients with favorable and unfavorable outcome were compared. In a logistic regression model adjusted for age, sex and Glasgow Coma Scale on admission, a CPP greater than 75 mmHg was marginally statistically significantly related to outcome at 6 months after injury.

**Conclusions:**

Patients with favorable outcome had certain common features in terms of microdialysis parameters and CPP values. An individualized approach regarding CPP levels and cut -off points for Glycerol concentration and L/P ratio are proposed.

## Background

Traumatic Brain Injury (TBI) is a major cause of death and disability [1]. Beyond the primary injury, the secondary insults account for an unfavorable outcome [2]. It is now feasible to monitor and record physiological variables with computerized multimodality monitoring systems in the neurointensive care unit patients. Monitoring assists the physician to implement the appropriate therapy and gives information about the expected outcome [3,4]. It has been shown that poor outcome is related to high Intracranial Pressure (ICP), and low Cerebral Perfusion Pressure (CPP) [5-7]. Besides that, brain metabolic status, as it is expressed via glucose, glycerol and lactate-pyruvate (L/P) ratio has also been correlated to clinical outcome in various studies. High values of L/P ratio, Glycerol concentration and PbtO_2_, as well as low levels of Glucose have been related to poor outcome. However, data regarding certain cut-off points for the latter parameters are insufficient. Only broad value ranges for metabolic parameters are currently in use. The clinical use of CPP is based on theoretical suggestions that maintaining optimal cerebral blood flow is necessary to meet the metabolic needs of the injured brain [6]. The goal is to preserve the ischemic penubra and avoid exacerbation of secondary insults. Based on international guidelines, a low threshold of CPP value is recommended [8], while there is a controversy regarding the upper limit of CPP values [7]. It has been postulated that high values of CPP could be harmful, given that in many brain injuries brain auto-regulation is severely damaged. However, raised CPP values could be beneficial to many patients, even at relatively high ICP levels [9]. The aim of this paper was to investigate the relation of CPP values and clinical outcome in patients with severe brain injuries as well as to assess the effectiveness of multimodal brain monitoring in relation to patients outcome.

## Methods

Thirty - four patients (29 men and 5 women) participated in the study. They had suffered a traumatic brain injury and hospitalization in an intensive care unit was necessary (Glasgow Coma Scale ≤ 8). All patients received conservative treatment. Informed consent was obtained from the families of the patients. All patients were intubated, sedated and occasionally paralyzed. If an emergency operation was not required, cranial bolts for measurement of PbtO_2_, ICP and a brain MicroDialysis (MD) catheters were placed. Τhe three catheters were inserted into the brain through a triple lumen introducer kit (Integra, CMA 70 microdialysis bolt catheter) in the right frontal lobe, in the case of a diffuse damage, or in the ipsilateral frontal lobe in the case of a regional damage. A networked computerized multimodality monitoring system was used. Microdialysis data were collected every 2 hours and ICP and CPP were studied. Brain Glucose (Glu), Glycerol (Gly), Pyruvate, Lactate concentrations and PbtO_2 _were monitored. All patients were treated according to modern neurointensive care principles. The goal was to maintain a level of ICP < 20 mmHg, an acceptable PbtO_2 _~ 20 mm, a Lactate/Pyruvate (L/P) ratio around 20-25, and definitely below 40. CPP values were appropriately adjusted, among other neuroprotective interventions, in order to achieve the aforementioned objective. The brain monitoring commenced within the first 24 hours of brain injury and continued until catheters were removed due to normalization of the monitored parameters without any neuroprotective intervention for 24 hours. For homogeneity reasons, in the case of prolonged monitoring, data from the first 10 days of hospitalization were evaluated. Glasgow Outcome Scale was used to evaluate patient outcome at 6 months after the injury. Ethical approval was given with the 158-22711 decision of the local ethical committee ("Koutlimpaneio & Triantafyllio Hospital" Scientific Committee).

## Statistics

The Mann-Whitney U-test was used to compare median values of variables that were not normally distributed (Normality control test: Shapiro-Wilk). GOS variable was dichotomized (binary distribution) according to favorable and unfavorable outcomes. Values greater than 3 (> 3) were considered as favorable outcome, while values ≤3 as an unfavorable one.

Mean CPP values were dichotomized at the level of 75 mmHg. The *x*^2 ^test was used for comparisons of outcome rates, and Fisher's exact probability test was used, when the number of observations in a 2 × 2 contingency table was small enough. A logistic regression model was used for outcome prognosis. SPSS 17.0 was used for statistics.

## Results

Demographic characteristics of the sample, evaluation data on admission and outcome are shown in Table 1. Twenty nine patients were men and 5 women. Mean age was 43.6 yrs ± 18.70. Median GCS value on admission was 7. 15 patients had a favorable prognosis at 6 months, 19 patients had an unfavorable one, whereas 14 patients of the latter group died (Mortality rate 41.2%) (Table 1). Differences in microdialysis markers and CPP/ICP values between the two groups of favorable and unfavorable outcome are shown in Table 2. Mean values of PbtO2, CPP, ICP and glucose concentration were higher in the group of favorable outcome, while L/P ratio and Glycerol concentration was lower. A statistically significant difference was observed when comparing the distributions of L/P ratio and glycerol in the two groups.

**Table 1:** Demographic characteristics of the sample, evaluation on admission and outcome

	Mean Value/Median (25th-75th)	SD	N
Patients age	43,6	18,7	34

Sex (men/women)			29/5

GCS score on admission	7 (6-8)		

Favorable outcome at 6 months			15 (44.1%)

Unfavorable outcome at 6 months			19 (55.9%)

Mortality rate			14/34 (41.2%)

**Table 2:** MD values in favorable and unfavorable outcome groups

			L/P	GLU	GLY	ICP	CPP	PbtO2
**Unfavorable outcome**	**N**		**19**	**19**	**19**	**19**	**19**	**19**
	
	Mean		31.86	1.12	80.68	20.42	84.58	29.51
	
	Median		34.17	1.00	66.10	16.00	84.00	28.30
	
	SD		8.33	0.61	50.46	14.65	8.87	5.91
	
	Min		13	0.11	25.92	6	70	20.45
	
	Max		46	2.75	216.80	60	98	37.70
	
	Percentiles	5	13.20	0.11	25.92	6.00	70.00	20.45
		
		25	25.39	0.78	51.29	12.00	77.00	24.01
		
		50	34.17	1.00	66.10	16.00	84.00	28.30
		
		75	38.18	1.36	103.98	23.00	94.00	34.84
		
		95	46.35	2.75	216.80	60.00	98.00	37.70

Favorable outcome	N		15	15	15	15	15	15
	
	Mean		24.10	1.57	38.64	27.20	78.53	32.33
	
	Median		24.03	0.91	33.18	20.00	82.00	29.03
	
	SD		6.88	1.46	13.19	18.21	23.05	14.93
	
	Min		13	0.41	24.86	13	75	17.56
	
	Max		37	5.07	71.48	82	103	80.29
	
	Percentiles	5	12.86	0.41	24.86	13.00	5.00	17.56
		
		25	18.13	0.62	30.31	17.00	72.00	22.58
		
		50	24.03	0.91	33.18	20.00	82.00	29.03
		
		75	30.18	2.71	42.10	29.00	89.00	36.47
		
		95	36.53	5.07	71.48	82.00	103.00	80.29
	
	p		0.007*	0.811**	0.000**	0.071**	0.248*	0.455*

*Mann-Whitney-U test

**T-test

**Figure 1:**
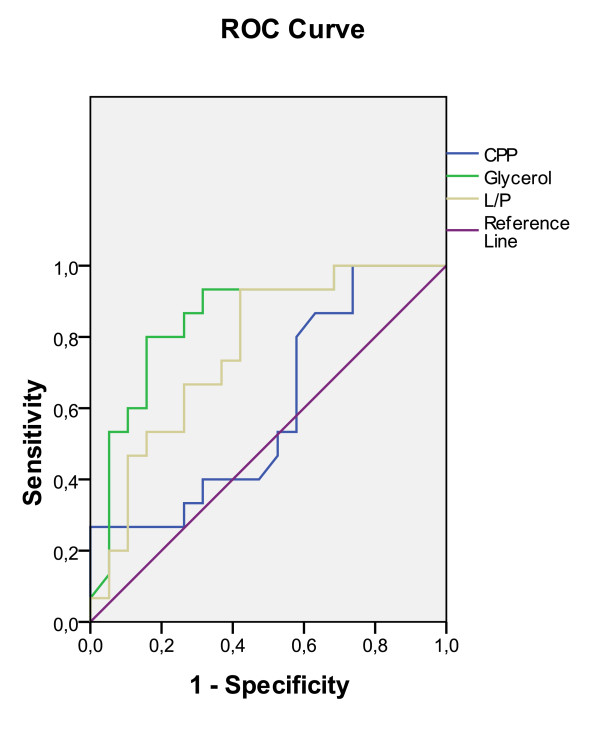
**ROCs for CPP, Glycerol and L/P values**.

The study of L/P and Glucerol concentration distributions revealed that no patient with favorable prognosis had either an L/P ratio or a Glycerol concentration greater than 37 and 72 mmol/l respectively. Regarding CPP distribution, no patient with a favorable prognosis had a CPP value lower than 75. Six patients with an unfavorable outcome had an L/P value and a Glycerol concentration lower than 37 and 72 mmol/l respectively. Five patients with an unfavorable outcome had a CPP value lower than 75 mmHg (Table 3). If the aforementioned critical values were regarded as cut-off points, meaningful statistical results were produced for L/P, CPP and glycerol values. A statistically significant difference was established between the favorable and unfavorable prognosis groups regarding L/P and Glycerol, while a marginal significance was found for CPP. It is noted, that all 15 patients with a favorable outcome had L/P ratio < 37, CPP ≥ 75 mmHg and glycerol < 72 mmol/l. ROCs for Glycerol, L/P and CPP are presented in Figure 1. Regarding the other parameters, 3 patients with favorable outcome had a Glucose concentration greater than 2.75, 3 patients had a PbtO_2 _greater then 37, while 1 patient had an ICP value greater than 60 mmHg (data not shown). The mean value of PbtO_2 _was slightly higher in patients with favorable outcome exhibiting a broader standard deviation. However, no statistically significant difference was observed between the two groups (favorable outcome group: 32.33 ± 14.93, unfavorable outcome group: 29.51 ± 5.91, F = 1.2, t = -0.17,*p *= 0.864). Variables that were statistically significantly related to outcome in the univariate analysis were included in a logistic regression model. After adjusting for age, sex and GCS on admission, the odds for the patients with a CPP greater than 75 mmHg were 11.1 higher for favorable outcome at 6 months after the injury (*p *= 0.052-Table 4).

**Table 3:** CPP, L/P ratio and Glycerol concentration in patients with favorable and unfavorable outcome

Parameters	Outcome		
	**Unfavorable**	**Favorable**	**Total**

CPP < 75 mmHg	5	0	5

CPP ≥ 75 mmHg	14	15	29

Total	19	15	34

Fischer's test, *p *= 0.053			

L/P > 37	6	0	6

L/P ≤ 37	13	15	28

Total	19	15	34

Fischer's test, *p *= 0.024			

Glycerol > 72 mmol/l	6	0	6

Glycerol ≤ 72 mmol/l	14	15	28

Total	19	15	34

Fischer's test, *p *= 0.024			

**Table 4:** Logistic regression model for outcome prognosis adjusted for age and gender

	B	**Sig**.	Odds Ratio	95% C.I.for Exp(B)	
				
				Lower	Upper
CPP ≥ 75 mmHg	-2.407	0.052	11.1	0.970	125.000

Glycerol > 72 μg/lt	0.247	0.867	1.280	0.071	23.192

Lp > 37	-2.583	0.202	0.076	0.001	3.998

Age	0.001	0.977	0.999	0.956	1.044

GCS	0.149	0.565	1.161	0.699	1.929

Gender (men = 1)	-1.995	0.117	0.136	0.011	1.653

Constant	2.029	0.428	7.605		

Dependent variable: Outcome.

Coding: Favorable outcome = 1, Unfavorable = 2, CPP ≥ 75 = 2, CPP < 75 = 1

## Discussion

According to the findings of the present study, certain cut-off values in microdialysis parameters are helpful to determine patients' outcome. Favorable outcome was also related to CPP values beyond the consensus range of 60-70 mmHg.

There has been a controversy about CPP upper limits in patients with severe head injuries [6,7,10,11]. A lower CPP goal of 60 mmHg has been endorsed by the American Association of Neurological Surgeons and recently the Brain Trauma Foundation suggested a general threshold in the realm of 50-70 mmHg [8]. In this study, CPP regulation was guided by brain biochemistry and emphasis was placed on clinical outcome, provided that L/P ratio and PbtO_2 _remained at an acceptable level. The sum of patients with a favorable prognosis raised with an increased CPP beyond 70 mmHg. The 75 mmHg finally turned out to be the critical value. Patients who survived and exhibited CPP values over 75 mmHg exhibited an excellent outcome as well. The explanation possibly lies in PbtO_2_. Stocchetti et al. had found that cerebral perfusion pressure augmentation significantly increased levels of brain tissue oxygen and significantly reduced the regional oxygen extraction fraction. Patients with baseline PbtO_2 _values at low levels had demonstrated a greater increase in PbO_2 _in response to CPP augmentation [12]. Values greater than 60 mmHg were also related to a more favorable PbtO_2 _in the study of Caballos et al. [13]. Similar findings have been reported by Johnston et al. [14] and other researchers. Rosner and colleagues have studied the maintenance of a CPP greater than 70 mmHg in patients with severe TBI. Their results were excellent, with an overall mortality rate of 21%. Fifty-four percent of survivors had a good recovery or moderate disability [15]. In their recent study, Nelson et al. demonstrated that patients with traumatic brain injury and a CPP in the realm of 70 mmHg exhibited better prognosis, although no significant relation was found between outcome and MD and MD values were poorly related to CPP [16].

Regarding L/P ratio and Glycerol, existing data also support our findings. Hutchinson et al. measured an L/P value of 26.38 ± 8.1 in patients with a traumatic brain injury, without signs of ischemia using PET (Positron Emission Tomography), with a normal Oxygen Extraction Fraction (OEF was approximately 0.4) [17]. In a study including 21 patients who underwent surgery for a hematoma caused by spontaneous intracerebral hemorrhage, it was found that the zone surrounding the evacuated hematoma area exhibited a biochemical behavior similar to that of the zone surrounding the brain contusion, with an increase in the value of the lactic-pyruvate ratio of about 35 and an increase in glycerol concentration [11]. That fact can be helpful in making a decision, whether the injury should be treated surgically or not.

Regarding Glycerol concentration, our data are in accordance with those of Peerderman et al., which measured glycerol levels in 15 patients with traumatic brain injury. Patients with an unfavorable prognosis had higher glycerol levels during the first 24 h of hospitalization. They also noticed that no patient with favorable prognosis had a glycerol concentration greater than 150 μmol/L, while 6 out of 10 patients with an unfavorable prognosis had one. [18]. Clausen at al also observed significantly increased glycerol concentrations when PbtO_2 _was lower than 10 mm Hg or when cerebral perfusion pressure was lower than 70 mm Hg in TBI patients [19].

The low GCS on admission could account for the relatively high mortality in the present study. Depending on severity, TBI mortality rates within a 6 month period after the injury, ranged from 3% to 35% [20]. The relationship between TBI severity at onset and mortality is well documented [21,22]. It is noticed, however, that our data refer to adults below 65 years old, suffering from severe TBIs, who were administered conservative treatment only. A model including as many clinical and laboratory parameters as possible and a large number of patients with specific and homogenous brain damage features is necessary for definite conclusions to be drawn.

## Conclusion

The findings of this study support the maintenance of high CPP levels guided by the metabolic status of the brain tissue and suggest certain cut-off values for microdialysis parameters. The findings support the latest BTF guidelines, which suggest a more individualized approach in CPP regulation. However, optimal CPP values may vary from patient to patient and over time, as the physiological environment of the injured brain changes. Neurointensivists should not be discouraged to maintain high CPP levels, if the brain metabolic state allows so. We suggest a broader monitoring including PbtO2, microdialysis, and local cerebral blood flow measure. Interventions should be guided by each patient's brain metabolic status, aiming for the best possible outcome.

## Authors' contributions

PT: study design, data collection, writing; PK: critical review, writing, study design; FK: critical review, writing, study design; PG: study design, data collection; CA: study design, data collection; TA: data collection; MM: data collection; PD: data collection; KA: editing, critical review; KA: study design, critical review; All authors read and approved the final manuscript.

## Conflict of interest

The authors declare that they have no competing interests.
